# Autologous cryo-shocked neutrophils enable targeted therapy of sepsis via broad-spectrum neutralization of pro-inflammatory cytokines and endotoxins

**DOI:** 10.3389/fchem.2024.1359946

**Published:** 2024-02-21

**Authors:** Qiuxia Gao, Yan Yan, Jie Zhang, Xiaoxue Li, Jiamei Wang, Yi Feng, Peiran Li, Huanhuan Wang, Yunlong Zhang, Lingjie He, Zhiyan Shan, Bin Li

**Affiliations:** ^1^ School of Inspection, Ningxia Medical University, Yinchuan, Ningxia, China; ^2^ Institute of Translational Medicine, The First People’s Hospital of Foshan, Foshan, Guangdong, China; ^3^ Molecular Diagnosis and Treatment Center for Infectious Diseases, Dermatology Hospital of Southern Medical University, Guangzhou, Guangdong, China; ^4^ Department of Critical Care Medicine, Department of Emergency, Renmin Hospital of Wuhan University, Wuhan, Hubei, China; ^5^ Engineering Research Center of Tibetan Medicine Detection Technology, Ministry of Education, Xizang Minzu University, Xianyang, Shaanxi, China; ^6^ Department of Histology and Embryology, Harbin Medical University, Harbin, Heilongjiang, China

**Keywords:** sepsis, cytokine storm, endotoxin, neutrophils, cryo-shock, broad-spectrum anti-inflammatory

## Abstract

**Background:** Sepsis is a life-threatening disease characterized by multiple organ failure due to excessive activation of the inflammatory response and cytokine storm. Despite recent advances in the clinical use of anti-cytokine biologics, sepsis treatment efficacy and improvements in mortality remain unsatisfactory, largely due to the mechanistic complexity of immune regulation and cytokine interactions.

**Methods:** In this study, a broad-spectrum anti-inflammatory and endotoxin neutralization strategy was developed based on autologous “cryo-shocked” neutrophils (CS-Neus) for the management of sepsis. Neutrophils were frozen to death using a novel liquid nitrogen “cryo-shock” strategy. The CS-Neus retained the source cell membrane structure and functions related to inflammatory site targeting, broad-spectrum inflammatory cytokines, and endotoxin (LPS) neutralizing properties. This strategy aimed to disable harmful pro-inflammatory functions of neutrophils, such as cytokine secretion. Autologous cell-based therapy strategies were employed to avoid immune rejection and enhance treatment safety.

**Results:** In both LPS-induced sepsis mouse models and clinical patient-derived blood samples, CS-Neus treatment significantly ameliorated cytokine storms by removing inflammatory cytokines and endotoxin. The therapy showed notable anti-inflammatory therapeutic effects and improved the survival rate of mice.

**Discussion:** The results of this study demonstrate the potential of autologous “cryo-shocked” neutrophils as a promising therapeutic approach for managing sepsis. By targeting inflammatory organs and exhibiting anti-inflammatory activity, CS-Neus offer a novel strategy to combat the complexities of sepsis treatment. Further research and clinical trials are needed to validate the efficacy and safety of this approach in broader populations and settings.

## 1 Introduction

Sepsis is defined as a dysregulated host inflammatory response to infection (bacterial, fungal, or viral) (Goldfarb et al., 2002; Harriett et al., 2022; Tian et al., 2023) that is often life-threatening and is the leading cause of death in the intensive care unit (ICU) (Angus and van der Poll, 2013). One of its hallmarks is an over-activated immune response and systemic inflammatory response syndrome (SIRS), which, in severe cases, can lead to a “cytokine storm” (Huang et al., 2019). Sepsis precipitates a collapse of biological function and leads to tissue damage and multiple organ dysfunction even failure, namely, multiple organ dysfunction syndrome (MODS) (Critical Care Medicine, 1992). The number of hospitalizations due to sepsis continues to increase despite many efforts to find effective treatment procedures (Cohen et al., 2015). Endotoxin (*i.e.*, lipopolysaccharide [LPS]) released by Gram-negative bacteria is an important pathogenic trigger of sepsis and is the main cause of SIRS, which is characterized by the production of large amounts of pro-inflammatory cytokines, causing fever and intravascular coagulation and ultimately leading to septic shock (Angus and van der Poll, 2013). Evidence suggests that the spread of LPS from the site of infection to the whole body is the key pathogenetic mechanism that leads to this dramatic immune imbalance (Leentjens et al., 2013; Ronco et al., 2023). Since higher levels of endotoxin are associated with worsened clinical outcomes, effective removal of endotoxin is a key element in the successful treatment of sepsis (Davies and Cohen, 2011; Esteban et al., 2013; Ronco et al., 2023). On the other hand, the “cytokine storm” encompasses a large number of pro-inflammatory cytokine releases and interactions, and the removal of only one cytokine cannot break the integrity of this network. Thus, there is an urgent need to develop broad-spectrum cytokine removal strategies.

Clinically, traditional treatments have relied on antibiotics and supportive care (*e.g.*, fluid resuscitation and mechanical ventilation) (Vincent, 2022) but have been ineffective, with mortality rates remaining as high as 50% of treated patients (Brown and Semler, 2019). Neutralization and elimination of endotoxins face various challenges. Antibiotics that are effective in neutralizing endotoxins (*e.g.*, polymyxins) have limited their clinical application because of their strong nephrotoxicity and neurotoxicity (Esteban et al., 2013; Pickkers and Russell, 2019). The attachment of these molecules to solid-phase carriers for blood perfusion maintains their endotoxin-binding properties while minimizing toxic effects, but evidence of clinical efficacy has not been established (Dellinger et al., 2018; Pickkers and Russell, 2019). As for pro-inflammatory cytokine elimination, the development of the monoclonal anti-tumor necrosis factor (TNF) antibody F(ab')2 fragment afelimomab significantly reduces circulating levels of TNF-alpha and interleukin-6 (IL-6) and slightly prolongs survival in sepsis patients with elevated IL-6 (Gallagher et al., 2001). Nevertheless, the efficacy of afelimomab is unstable.

Neutrophils (Neus) are the most abundant immune cells in the blood, accounting for 50%–70% of all leukocytes. Like macrophages, Neus have multiple Toll-like receptors (e.g., TLR2 and TLR4) (Li et al., 2021; Zhang et al., 2023) and pro-inflammatory cytokine receptors (e.g., IL-1R and TNF-αR) (Kilpatrick et al., 2004; Saul-McBeth et al., 2021) that are highly expressed on their cell membranes. Notably, TLR4 is the specific receptor for LPS and can directly and potently adsorb LPS (Thamphiwatana et al., 2017; Ciesielska et al., 2021). Cell membrane mimetic nanoparticles prepared with Neus membranes can precisely target inflammatory foci and broadly neutralize pro-inflammatory cytokines, leading to the effective treatment of rheumatoid arthritis (Zhang et al., 2018).

Considering the simplicity of obtaining blood and extracting Neus from it (Zhao et al., 2019), coupled with the abundance of Neus and its potential neutralization ability of LPS and broad-spectrum anti-inflammatory effects, the use of autologous Neus as raw materials for the treatment of sepsis is theoretically feasible. However, live Neus are characterized by the release of pro-inflammatory cytokines and inflammatory infiltration (Shen et al., 2017), thus requiring Neus to be lethal but retaining its intact cell membrane structure and function.

**SCHEME 1:**
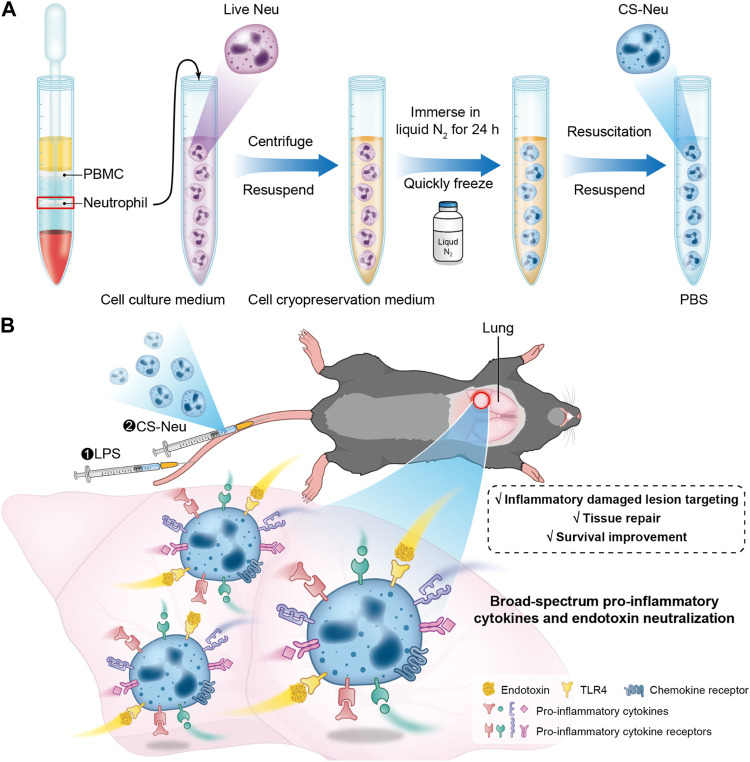
Schematic illustration of the preparation route, the sepsis animal model establishment process, and the treatment mechanism of CS-Neus. **(A)** Schematic diagram of preparation of CS-Neus. **(B)** Therapeutic mechanism of CS-Neus in an LPS-induced sepsis mouse model.

In this contribution, we developed a novel “cryo-shock” strategy that allows liquid N_2_ to freeze Neus to ensure complete death and loss of its pro-inflammatory effects while preserving its cell membrane structure and associated receptor functions. In detail, fresh Neus were suspended in a non-controlled-rate cell cryopreservation medium and immersed into liquid N_2_ for 24 h to ensure cell death. Next, cryo-shocked Neus (CS-Neus) were obtained after standard cell resuscitation procedures (Scheme 1A). We hypothesized that compared with heat/chemical-induced cellular death, the cryo-shock process does not trigger protein denaturation but retain the receptors on the cell membrane. In a mouse model of LPS-induced sepsis (Ye et al., 2022), the intravenous injection of CS-Neus precisely targets inflammatory damaged organs such as the lung due to the chemotaxis of these agents by inflammatory lesions. At the same time, CS-Neus can use TLR4 to directly neutralize LPS in plasma and tissues and organs and remove endotoxin. Then, CS-Neus that arrived at the lesions would utilize multiple pro-inflammatory cytokine receptors on their surface to broadly neutralize varied pro-inflammatory cytokines, thus achieving anti-inflammatory potential. More importantly, this strategy based on autologous Neus avoids the risk of immune rejection and therefore holds huge potential for clinical translation.

## 2 Results

### 2.1 Preparation and characterization of CS-Neus

**FIGURE 1:**
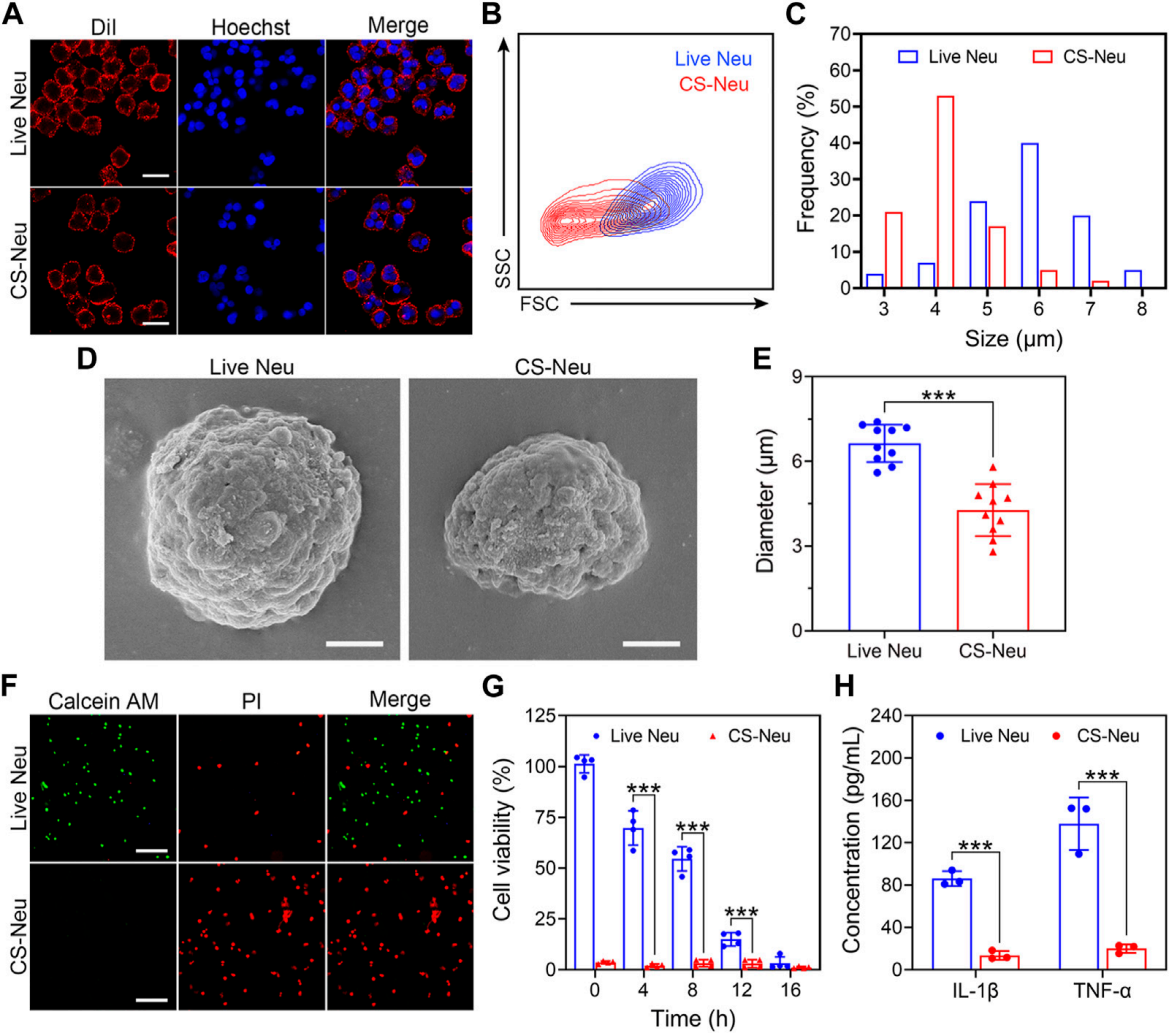
Characterization of CS-Neus. **(A)** Cell membrane integrity analysis of CS-Neus. Cell membranes were stained with the DiI dye (red), and cell nuclei were stained by Hoechst (blue). Live Neus served as a control. Scale bars: 10 μm. **(B)** Flow cytometry analysis of live Neus and CS-Neus using the same voltage. FSC, forward scatter; SSC, side scatter. **(C)** Size distributions of live Neus and CS-Neus. The cellular sizes were measured in **(A)** via the software program Nano Measurer (cell numbers = 100). **(D)** Typical SEM images of live Neus and CS-Neus. Scale bars: 2 μm. **(E)** Diameter comparison between live Neus and CS-Neus, as described in **(D)** (*n* = 10). **(F)** Live (Calcein AM)/dead (PI) fluorescence staining of live Neus and CS-Neus. Scale bars: 100 μm. **(G)** Cell viability analysis of live Neus and CS-Neus by CCK-8 assay (*n* = 4). **(H)** Comparison of the pro-inflammatory cytokine release capacity between live Neus and CS-Neus (*n* = 3). Data were presented as mean ± SD. Statistical significance was calculated through ordinary one-way analysis of variance (ANOVA). ***p < 0.001.

The “cryo-shock” technique proposed in this work is originated from a traditional cell cryopreservation process (Ci et al., 2020), which includes three steps: 1) suspension of cells in a non-controlled-rate cell cryopreservation medium; 2) placement of the resulting cell suspension in an −80°C refrigerator overnight; and 3) immersion of the cell suspension into liquid N_2_ for prolonged storage. During these steps, leaving the cell suspension at −80°C overnight (Step 2) serves as an important temperature buffer with the purpose of preventing cell death due to direct exposure to liquid N_2_. The “cryo-shock” procedure in this study removed this step, allowing the neutrophils to be frozen to death to form a novel type of dead cell carrier (CS-Neu). We hypothesized that CS-Neus were completely dead after “cryo-shock” treatment and eliminated the inflammation-causing side effects of live Neus but still maintained an intact cellular structure and functional receptors on their cell membrane, including chemokine receptors, pro-inflammatory cytokine receptors, and Toll-like receptors, which help realize precise tracking of inflammatory foci, broad-spectrum neutralization of inflammatory cytokines, and removal of endotoxins (LPS). As shown in Figure 1A, the cell membrane and nuclei of CS-Neus were as intact as those of live Neus, indicating that the cellular structure is preserved. In addition, the forward scatter (FSC) value of flow cytometry showed that CS-Neus were reduced in size compared to live Neus, while the side scatter (SSC) value of CS-Neus was almost the same as that of living cells, suggesting that their intracellular structure remained unchanged (Figure 1B). Similarly, the diameter distribution statistics show a significant reduction in the size of the CS-Neus compared to the live Neus (Figure 1C). To visualize the sizes and structures of CS-Neus more intuitively, scanning electron microscopy (SEM) was employed. Compared to live Neus, which were spherical in shape, CS-Neus exhibited notable volume shrinkage and increased roughness of the cell surface but retained their spherical shape, suggesting that their biological functions may be preserved (Figure 1D). Moreover, statistical analysis of cell diameters in the SEM images showed the same trend as the cell sizes, as shown in Figures 1B, C, *i.e.*, the diameters of CS-Neus were remarkably smaller than those of live Neus (6.64 μm vs. 4.27 μm) (Figure 1E).

Next, we investigated the effect of the “cryo-shock” technique on the cell viability of Neus by live (Calcein AM)/dead (PI) fluorescent staining and Cell Counting Kit 8 (CCK-8) assay. As illustrated in Figure 1F, compared to live Neus (most cells were stained by Calcein AM), 100% of CS-Neus were stained by PI, indicating complete cell death. Similarly, CCK-8 assay revealed a complete loss of cell viability of CS-Neus, as compared with live Neus (Figure 1G). More importantly, CS-Neus did not release pro-inflammatory cytokines (IL-1β and TNF-α) in response to LPS stimulation compared to live Neus, which demonstrated that their inflammation-causing activities were effectively inhibited (Figure 1H).

Collectively, CS-Neus obtained by “cryo-shock” treatment retained the cellular morphology, cell membranes, and nuclei of live Neus, although their sizes were reduced. What counts is these CS-Neus lost side effects such as the release of inflammatory cytokines and could be applied as a high-performance dead cell formulation for the treatment of sepsis.

### 2.2 Biosafety assessment of CS-Neus

**FIGURE 2:**
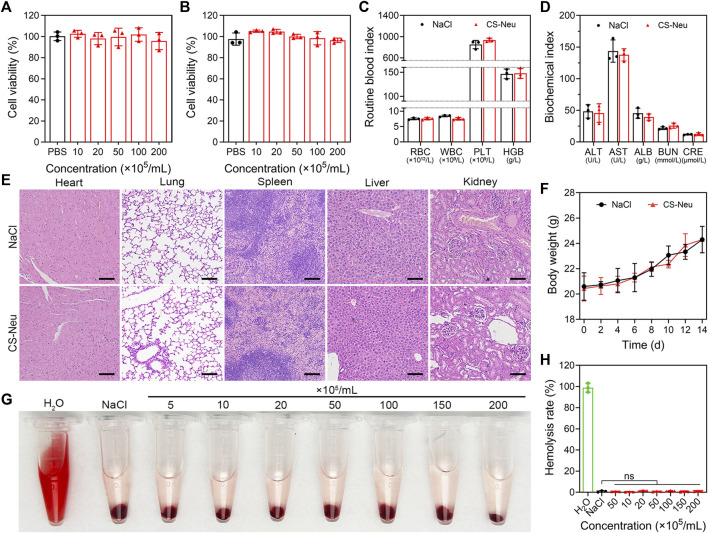
Biotoxicity evaluation of CS-Neu. **(A,B)** Cell viability analysis of HUVEC and L929 cells after coincubation with different concentrations of CS-Neu for 24 h (CCK-8 assay). **(C,D)** Routine blood tests and plasma biochemical parameter detection of healthy mice 24 h after intravenous injection of 250 μL of the CS-Neu suspension (4.0 × 10^7^ count mL^−1^) or NaCl. **(E)** HE staining analysis of varied organs isolated from healthy mice 24 h after intravenous injection of 250 μL of the CS-Neu suspension (4.0 × 10^7^ count mL^−1^) or NaCl. Scale bars: 100 μm. **(F)** Body weight monitoring of healthy mice after intravenous administration of 250 μL of the CS-Neu suspension (4.0 × 10^7^ count mL^−1^) or NaCl. **(G,H)** Hemolysis-promoting ability of CS-Neu (prepared from healthy human neutrophils) with different concentrations after coincubation with fresh human erythrocytes (isolated from the same volunteers) for 24 h at 37°C. NaCl was set as negative control; H_2_O was set as positive control. Data were presented as mean ± SD. Statistical significance was calculated through ordinary one-way analysis of variance (ANOVA).

Biosafety and biocompatibility analysis of CS-Neus is a prerequisite for realizing its *in vitro* and *in vivo* applications. On the other hand, CS-Neus is derived from autologous Neus and theoretically has no risk of immune rejection, but it is essential to explore its ability to trigger immune rejection. Here, we evaluated the biotoxicity and immune rejection potential of CS-Neus by co-incubation with normal cells, intravenous injection into healthy mice, and conducting hemolysis assay. As shown in Figures 2A, B, after 24 h of co-incubation with normal cells (HUVEC and L929 cell lines), CS-Neus did not impair the viability of these cells, even at a very high concentration (2.0 × 10^7^ counts mL^−1^). After injecting a high concentration of CS-Neus (200 μL, 2.0 × 10^7^ mL^−1^) intravenously into healthy mice, we analyzed routine blood indices, plasma biochemical indices, histopathological alterations in different vital organs, and body weight changes in these mice. As illustrated in Figure 2C, 24 h after injection of CS-Neus, the counts of red blood cells (RBC), white blood cells (WBC), platelets (PLT), and the concentration of hemoglobin (HGB) in the peripheral blood of the mice did not show apparent changes compared with the NaCl-treated group, and all of them were located in the normal range, which suggested that CS-Neus did not lead to damage to the hematopoietic and blood system of healthy mice. Of note, the fact that the counts of WBCs remained at normal levels indirectly proved that CS-Neus would not trigger immune rejection responses, which would induce obvious changes in neutrophils and lymphocytes (the primary components of WBCs). Additionally, the detection of plasma biochemical indices showed that the administration of a high concentration of CS-Neus for 24 h did not result in significant abnormalities in albumin (ALB), aminotransferase (ALT), aspartate aminotransferase (AST), blood urea nitrogen (BUN), and creatinine (CRE), suggesting that CS-Neus do not lead to hepatic and renal dysfunctions (Figure 2D). Subsequently, different vital organs from these mice were isolated for histopathological analysis. As shown by hematoxylin and eosin (H&E) staining, the administration of a high concentration of CS-Neus for 24 h did not cause severe inflammatory infiltration and substantial tissue damage in these organs, which was in accordance with the NaCl-administered group (Figure 2E). Furthermore, body weight monitoring of these mice showed no significant change in the body weight of the mice in the CS-Neus-administered group compared to the NaCl-treated group (Figure 2F).

We then evaluated the hemolysis ability of CS-Neus after co-incubation with fresh RBCs harvested from healthy humans. As shown in Figures 2G, H, different concentrations of CS-Neus did not induce hemolysis of healthy human erythrocytes after prolonged (24 h) co-incubation at 37°C, suggesting that CS-Neus were not toxic to RBCs. Notably, this result indirectly demonstrated that CS-Neus do not cause hemolysis caused by immune rejection.

Altogether, CS-Neus had a high biocompatibility and low risk of immune rejection and thus had potential for clinical promotion.

### 2.3 Chemotactic, anti-inflammatory, and endotoxin scavenging properties of CS-Neus *in vitro*

**FIGURE 3:**
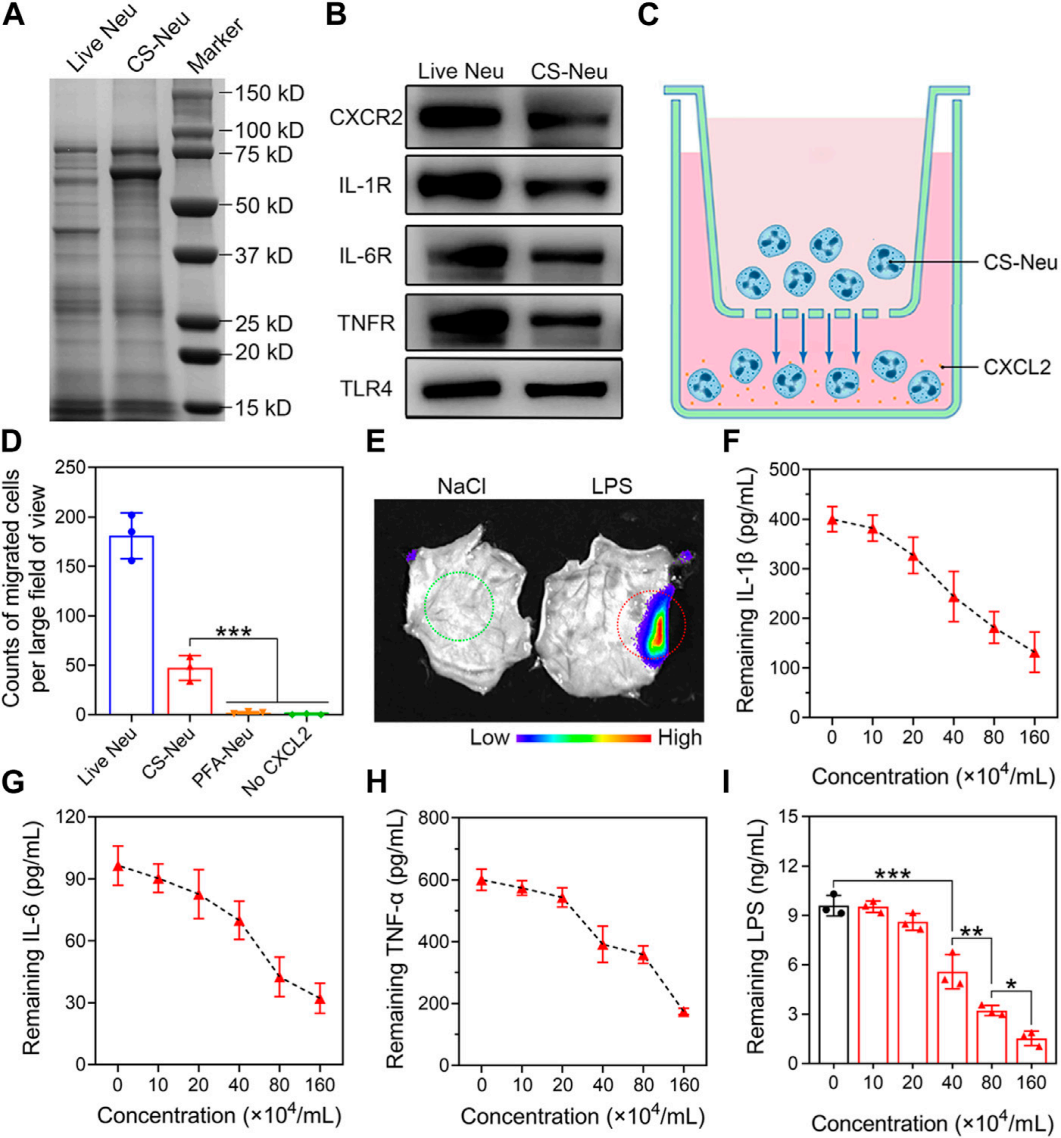
Chemotactic, anti-inflammatory, and endotoxin scavenging properties of CS-Neus. **(A)** SDS-PAGE analysis of live Neus and CS-Neus. Samples were run at equal protein concentrations. **(B)** Western blotting analysis of the chemokine receptor (CXCR2), varied pro-inflammatory cytokine receptors (IL-1R1, IL-6R, and TNFR), and TLR4 in live Neus and CS-Neus. **(C)** Schematic representation of the transwell assay showing CS-Neus chemotaxis by the chemokine CXCL2. **(D)** Counting the number of different types of cells that cross the well and migrate to the lower chamber of the transwell under the chemotaxis of CXCL2. The transwell system was placed at 37°C for 6 h. PFA-Neu, 4% paraformaldehyde induced dead Neus. **(E)** Fluorescence image of a piece of skin isolated from the back of a mouse 6 h after intravenous injection of CS-Neus (DiI-tagged, 1 × 10^7^ count mL^−1^, 100 μL). Six hours before CS-Neus administration, 20 μL of NaCl or 300 μg mL^−1^ of LPS solution (to cause local subcutaneous inflammation) was injected subcutaneously into the left (green circles) or right (red dashed circle) site of the mouse back, respectively. **(F–H)** Concentration-dependent neutralization of different pro-inflammatory cytokines by CS-Neus after co-incubation at 37°C for 6 h. **(I)** Concentration-dependent neutralization of LPS by CS-Neus after co-incubation at 37°C for 6 h. Data were presented as mean ± SD (*n* = 3). Statistical significance was calculated via one-way ANOVA **(D,I)**. *p < 0.05, **p < 0.01, and ***p < 0.001.

It has been reported that Neus inherently express various chemokine receptors (such as CXCR2 and CXCR3) (Chami et al., 2017; Adrover et al., 2019), pro-inflammatory cytokine receptors (such as IL-1β, IL-6, and TNF-α receptors) (Scapini et al., 2000; Akgul and Edwards, 2003; Croxford et al., 2014), and Toll-like receptors (such as TLR4) (Chakravarti et al., 2009; Janke et al., 2009; García-Culebras et al., 2019) on their cell membrane. In addition, cell membrane nanoparticles prepared from Neus membranes achieved efficient anti-inflammatory treatment of rheumatoid arthritis through broad-spectrum pro-inflammatory cytokine adsorption and elimination. In the present work, we hypothesized that CS-Neus obtained by “cryo-shock” treatment retains these biological receptors, as well as receptors’ conformations and functions, and can function in inflammatory chemotaxis, broad-spectrum neutralization of pro-inflammatory cytokines, and efficient endotoxin clearance. Therefore, we initially detected the expression of these functional receptors by sodium dodecyl sulfate–polyacrylamide gel electrophoresis (SDS-PAGE) and Western blotting tests. As shown in Figure 3A, the protein bands of live Neus and CS-Neus were almost identical, indicating that the major protein components of Neus were preserved after the “cryo-shock’ treatment. Importantly, the results of the Western blotting experiments showed that the protein expression of chemokine receptors (*e.g.*, CXCR2), pro-inflammatory cytokine receptors (including IL-1R1, IL-6R, and TNFR), and Toll-like receptors (*e.g.*, TLR4) was preserved on CS-Neus, predicting that their functions also remain present (Figure 3B).

To validate the chemotaxis of CS-Neus, we performed the transwell experiments (Figure 3C). As illustrated in Figure 3D, a large number of live Neus and CS-Neus entered the lower chamber of the transwell under the chemokine CXCL2 chemotaxis, indicating that the chemotaxis of CS-Neus was preserved. Notably, Neus that died after treatment with paraformaldehyde completely (known as PFA-Neus) lost chemotaxis because the chemical toxicity of paraformaldehyde disrupted the function of the chemokine receptor and/or rendered the cells deformable. In addition, CS-Neus do not enter the lower lumen in the absence of CXCL2, confirming the chemotaxis of CS-Neus as belonging to chemotaxis toward inflammation. These results make it possible for CS-Neus to penetrate the capillaries and enable precise tracking of inflammatory lesions. To further explore the inflammatory chemotaxis of CS-Neus *in vivo*, we established a mouse model of LPS-induced local subcutaneous inflammation. As depicted in Figure 3E, intravenously injected CS-Neus (DiI-labeled) selectively targeted LPS-induced foci of skin inflammation (red-circled sites), whereas little targeting was observed at the green-circled sites of localized subcutaneous injections of NaCl (no inflammation was triggered). Next, we assessed the role of CS-Neus in the broad-spectrum neutralization of pro-inflammatory cytokines *in vitro* by the co-incubation of CS-Neus with different pro-inflammatory cytokines. Mechanistically, neutralization of pro-inflammatory cytokines is achieved by receptor–ligand-specific binding. As shown in Figures 3F–H, CS-Neus have a typical concentration-dependent pro-inflammatory cytokine neutralization ability toward IL-1β, IL-6, and TNF-α. Furthermore, CS-Neus likewise showed a concentration gradient-dependent LPS removal effect, in addition to the adsorption of LPS by their TLR4 receptors on the cell membrane (Figure 3I).

Altogether, CS-Neus present chemotaxis to sites of inflammation, broad-spectrum clearance of pro-inflammatory cytokines and endotoxins, and can be used as a promising biologic agent for the targeted therapy of sepsis.

### 2.4 Targeted therapeutic efficacy of CS-Neus for sepsis

**FIGURE 4:**
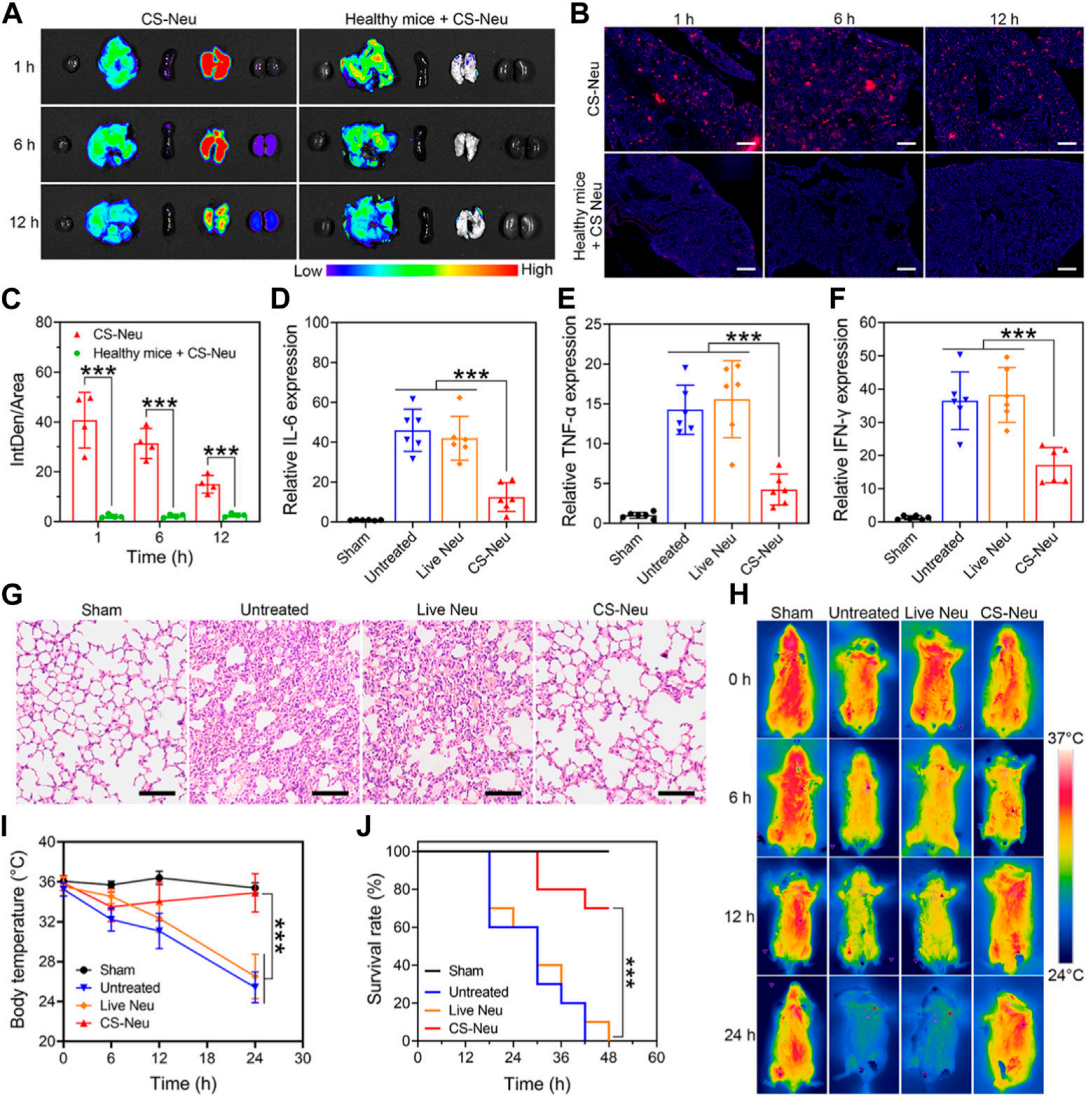
Focal targeting ability and comprehensive efficacy of CS-Neus for sepsis. **(A)** Fluorescence monitoring of different organs (left to right: the heart, liver, spleen, lung, and kidney) isolated from sepsis mice injected intravenously with 3.0 × 10^6^ count of DiI-tagged CS-Neus. Healthy mice intravenously injected with 3.0 × 10^6^ count of DiI-tagged CS-Neus were used as a control. **(B,C)** Frozen sections and their DiI fluorescence intensity analysis of lung tissues described in **(A)** (*n* = 4). The tissues were stained by DAPI. Scale bar: 500 μm. **(D–F)** Relative expression of different pro-inflammatory cytokines in lung tissues of sepsis mice after different treatments (*n* = 6). Treatments: LPS-induced sepsis model was built at 0 h; at 2, 4, and 6 h, 9.0 × 10^6^ count of Live Neus or CS-Neus were injected intravenously; at 48 h, the mice were sacrificed, and lungs were isolated for analysis. **(G)** Histopathological assessment of the lungs of sepsis mice after different treatments described in **(D)**. Scale bars: 100 μm. **(H,I)** Time-dependent body temperature measurement of sepsis mice after different treatments described in **(D)** (*n* = 4). **(J)** Survival of sepsis mice after different treatments described in **(E)** (*n* = 10). Data were displayed as mean ± SD. Statistical significance was calculated through Student’s t-test **(C)**, ordinary one-way ANOVA **(D–F,I)**, or log-rank (Mantel–Cox) test **(J)**.***p < 0.001.

Subsequently, we evaluated the ability of CS-Neus to target inflammatory lesions in sepsis and their overall therapeutic efficacy. Sepsis is a dysregulated inflammatory response triggered by infection with pathogenic microorganisms such as bacteria (Font et al., 2020). LPS, one of the main components of bacteria, induces a strong immune response in immune cells, leading to the production of large amounts of pro-inflammatory cytokines and potentially triggering a cytokine storm (Faix, 2013; Moriyama and Nishida, 2021). Notably, intraperitoneal injection of LPS is a common method to build animal models of sepsis (Chen et al., 2022; Ye et al., 2022), which usually triggers inflammatory damages and dysfunctions in varied organs including the lung, kidney, intestine, and brain, especially the lung, sepsis shock, and eventually death (Gotts and Matthay, 2016; Moriyama and Nishida, 2021). Therefore, we first investigated the capability of CS-Neus to target the inflammatory damaged lungs. As illustrated in Figures 4A–C, intravenous injection of DiI-tagged CS-Neus (4.0 × 10^6^ count, 200 μL) presented obvious targeting to the inflammatory damaged lungs of sepsis mice, indicating the efficient chemotactic effect of CS-Neus on inflammatory lesions, which helps the targeted therapy of the disease. In contrast, CS-Neus could not realize the targeting of healthy lungs. Then, therapeutic assays were performed. Specifically, healthy mice were randomized into four groups, of which one was the sham group and the remaining three were the sepsis groups. At 0 h, an LPS-induced sepsis model was established. At 2, 4, and 6 h, the sepsis mice were untreated or treated with 9.0 × 10^6^ count of live Neus or CS-Neus. At 48 h, the mice were sacrificed, and lungs were isolated for analysis. As shown in Figures 4D–F, compared to the sham group, the expression levels of pro-inflammatory cytokines (such as IL-6, TNF-α and IFN-γ) were obviously elevated in the untreated group, which proved that the damaged lungs of septic mice were in a high-inflammatory state. Live Neus did not decrease the expression of these pro-inflammatory cytokines, indicating that they do not have anti-inflammatory effect, while CS-Neus showed significant capacity on reducing these cytokines, robustly proving their high-performance anti-inflammatory ability. H&E staining of lung tissues displayed that CS-Neus dramatically mitigated sepsis-induced inflammatory infiltration and tissue damage, verifying their tissue repair capability (Figure 4G).

Furthermore, the body temperature of sepsis mice from different groups exhibited that CS-Neus significantly inhibited the sepsis-induced body temperature decrease, which confirmed their overall efficient therapeutic outcomes (Figures 4H, I). More importantly, treatment with CS-Neus significantly increased the survival of septic mice, which proved that CS-Neu is a type of high-performance biologicals with precise targeting ability toward inflammation for the management of sepsis (Figure 4J).

### 2.5 Clearance pathways and clinical application of CS-Neus

**FIGURE 5:**
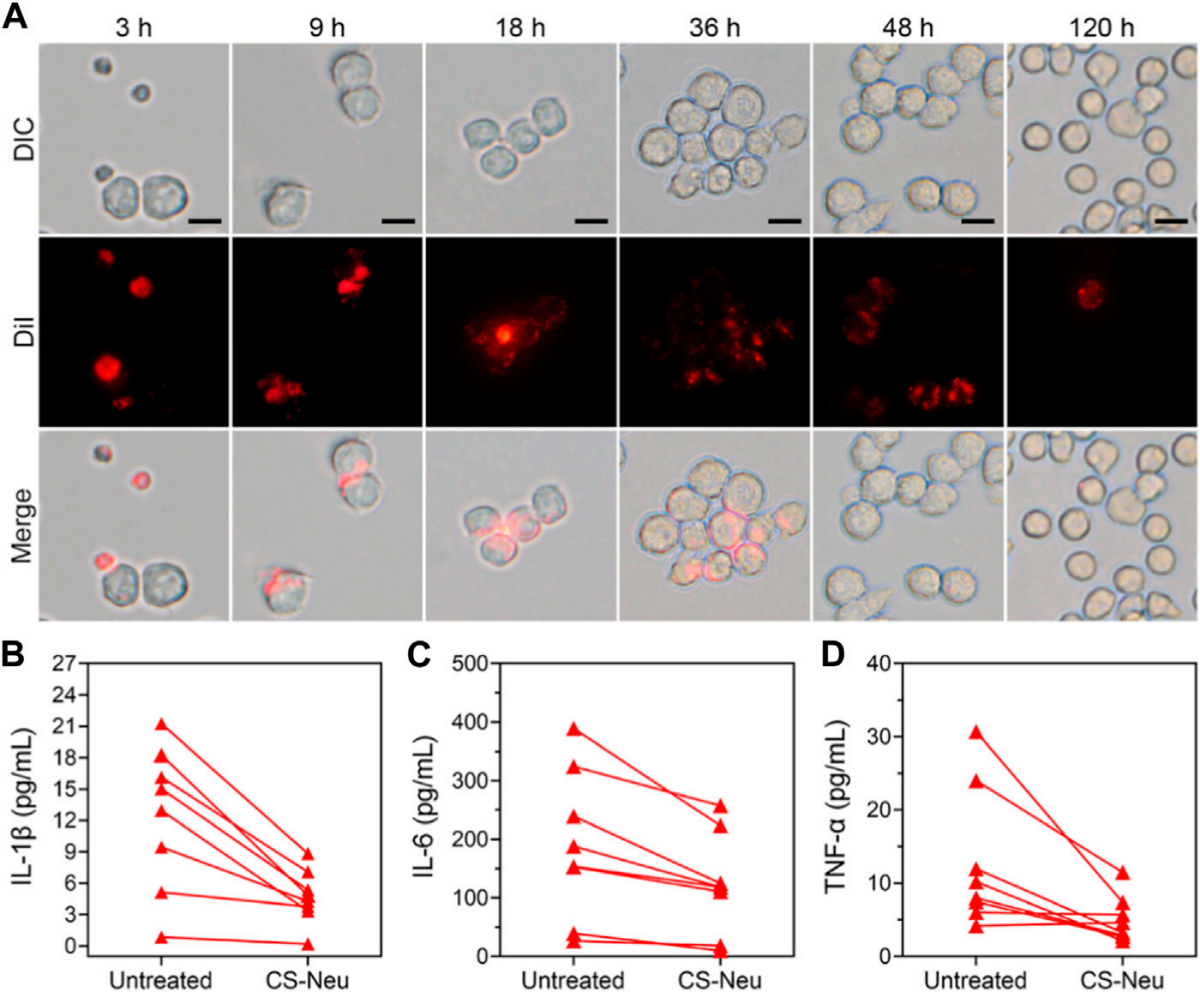
Clearance pathways and clinical application of CS-Neus. **(A)** Process of phagocytosis of DiI-tagged CS-Neus by macrophages (RAW264.7). Scale bars: 20 μm. **(B–D)** Concentrations of various pro-inflammatory cytokines in the plasma isolated from sepsis patients before and after co-incubation with CS-Neus (1.0 × 10^7^ count mL^−1^) at 37°C for 30 min. CS-Neus were fabricated from Neus isolated from the same patients.

Above results have shown that CS-Neus can efficiently adsorb and neutralize a variety of pro-inflammatory cytokines and LPS for the treatment of sepsis. We subsequently monitored the pathways through which CS-Neus was cleared by the host phagocytic system. Despite the fact that the raw material adopted to prepare CS-Neus is self-derived Neus (which avoids immune rejection), they are still a dead cell that can be recognized by the phagocytic system. Therefore, we hypothesized that CS-Neus would be engulfed and broken down by the host’s phagocytic system like common dead cells after performing their therapeutic functions. As shown in Figure 5A, CS-Neus (marked by DiI) were directly recognized and phagocytosed by macrophages after co-incubation at 37°C, and the phagocytosed CS-Neus were gradually degraded by macrophages over time, leading to structural disintegration, directly confirming the clearance pathway.

In order to facilitate the clinical translation of CS-Neus and to validate their clinical application value, we conducted experiments related to clinical samples. Specifically, plasma samples were collected from sepsis patients. At the same time, peripheral blood from these sepsis patients was also collected and Neus were isolated and used to fabricate CS-Neus. Finally, CS-Neus were incubated with the plasma of the same sepsis patients to determine their capacity in neutralizing and eliminating pro-inflammatory cytokines. As shown in Figures 5B–D, treatment of CS-Neus significantly reduced the concentrations of various pro-inflammatory cytokines (including IL-1β, IL-6, and TNF-α) in the patient’s plasma after 30 min of co-incubation at 37°C. This was attributed to the fact that various pro-inflammatory cytokine receptors, which were highly expressed on the surface of CS-Neus, efficiently adsorbed and neutralized these pro-inflammatory cytokines through the binding of the specific receptor ligands. These results confirmed that patient autologous-derived CS-Neus can be used to treat individual sepsis. This therapeutic strategy eliminates the risk of immune rejection (using foreign-derived cells) and therefore shows great potential for clinical application.

## 3 Discussion

Sepsis is a systemic inflammatory response caused by hyperactivation of the innate immune system caused by pathogen infection. It is one of the leading causes of death in the intensive care unit (ICU) and affects millions of patients each year globally. Notably, the complement system, the coagulation system, and the vascular endothelium are activated by the innate immune response, triggering a cytokine storm and subsequent organ dysfunction and failure, which are the main causes of death. Clinically, sepsis treatment is strongly dependent on antibiotics and supportive care, but mortality remains high. Thus, novel therapeutic strategies targeting the inflammatory drivers of sepsis are urgently needed.

Although current therapeutic studies aiming at the inflammatory response have achieved some success, there are still considerable limitations. Immunomodulatory imbalance in sepsis is coordinated by a complex network of molecules, and inhibition of just one or a few molecules may not be sufficient to prevent or reverse disease progression. Moreover, the toxicity of potent cytokine inhibition or antagonism remains highly unpredictable because of the wide range of pathways on which cytokines target. Accordingly, a single cytokine antagonist strategy not only cannot improve the survival rate of sepsis but also has serious side effects and safety risks.

Notably, tremendous progress has been made in the study of cell membrane biomimetic materials, offering a promising therapeutic platform. Natural membrane materials have been provided with high affinity and safety for the human body. Nonetheless, live cells are affected by the microenvironment *in vivo*, which inhibits the inherent function of cells. For example, neutrophils have superior targeting functions at inflammatory sites and neutralizing functions of broad-spectrum cytokines and endotoxins, while live neutrophils have disadvantages such as short life span, pro-inflammation, and neutrophil extracellular trap (NET) production. Consequently, current research is focused on eliminating the shortcomings of cell-derived biomimetic materials and further improving their functions by hybridizing with other materials, loading drugs, or gene editing to achieve better therapeutic effects.

Here, we subjected neutrophils to death by a novel “cryo-shock” strategy, which eliminated the pro-inflammatory function of neutrophils while preserving the membrane structure and function. The results indicated that CS-Neus had good cytokine and endotoxin-neutralizing effects, making it an excellent systemic anti-inflammatory effect. At the same time, it also opened up a new strategy for neutrophils in the field of anti-inflammatory therapy. Furthermore, CS-Neus retained a cell-like structure, suggesting its ability to load drugs for synergistic treatment. Alternatively, we found that the membrane of CS-Neu was brittle compared to that of living neutrophils, which was prone to fragmentation and generation of DNA tangles, leading to NETs. Therefore, this material needs to be further improved to maximize the function of neutrophils and eliminate the adverse factors.

In conclusion, based on the advanced “cryo-shock” technology, we have prepared cryo-shocked neutrophils with targeted broad-spectrum anti-inflammation and endotoxin neutralization for the treatment of sepsis, which is a hyperinflammatory disease. These CS-Neus retained functionalized chemokine receptors, pro-inflammatory receptors, and TLR-like receptors on their membranes, which have achieved remarkable anti-inflammatory effects and improved the survival rate of septic mice in *in vivo* and *in vitro* experiments. In particular, this strategy, on account of autologous cell modification and reinfusion, avoids the risk of immune rejection and toxic side effects. It provides a novel platform for the subsequent development of multifunctional bionic materials, which shows great potential for clinical application.

## 4 Materials and methods

### 4.1 Mice

Female ICR mice (8 weeks) were purchased from the Southern Medical University Laboratory Animal Center. All experiments involving animals were reviewed and approved by the Experimental Animal Ethics Committee of the Experimental Animal Center of Southern Medical University. Upon arrival, mice were given 1 week to adjust to the new animal facility prior to being used, maintained in a sterile environment, and allowed free access to food and water.

### 4.2 Materials

Lipopolysaccharides (LPS) were purchased from Sigma-Aldrich. NCR Cryopreservation Medium was purchased from Cyagen. Hoechst was ordered from Invitrogen. Cell culture-related reagents including Dulbecco’s modified Eagle’s medium (DMEM), fetal bovine serum (FBS), penicillin/streptomycin, and PBS were purchased from Gibco Life Technologies. Centrifuge tubes and glass bottom cell culture dishes were purchased from NEST Biotechnology Co., Ltd. The cell membrane red fluorescent staining kit (DiI) (cat. AC11038) was purchased from Shanghai Acmec Biochemical Co., Ltd. RIPA lysis buffer (cat: K1120) was obtained from APExBIO. Bicinchoninic acid (BCA) protein concentration test kit was purchased from KeyGEN Biotech. Calcein/PI cell viability/cytotoxicity assay kit, phenylmethylsulfonyl fluoride (PMSF), Coomassie brilliant blue, and Recombinant Murine MIP-2/CXCL2 were obtained from Beyotime Biotechnology. The neutrophil isolation solution kit, cell proliferation assay kit (CCK-8 assay) and dimethyl sulfoxide (DMSO) were obtained from Beijing Solarbio Science and Technology Co., Ltd. Rabbit anti-human IL-1R2, rabbit antihuman TNFR1, rabbit anti-human CXCR2, and rabbit anti-human TLR4 were obtained from Abcam. Rabbit anti-human IL-6Rα and anti-rabbit IgG secondary antibody were obtained from Proteintech. Goat anti-rabbit IgG-Fc secondary antibody (HRP) (cat. SSA003) was purchased from Beijing Sino Biological Inc. Goat Anti-Rabbit IgG (H + L) (cat. E-AB-1010) was purchased from Elabscience Biotechnology Co., Ltd. Mouse and human TNF-α, IL-1β, and IL-6 ELISA kits were purchased from Dakewe Biotech Co., Ltd. The Pierce Chromogenic Endotoxin Quant Kit was purchased from Thermo Fisher Scientific.

### 4.3 Cell culture

The murine macrophage cell line RAW 264.7, human umbilical vein endothelial cell line (HUVEC), and mouse fibroblast cell line (L929) were purchased from the American Type Culture Collection (ATCC). All cells were cultured in DMEM medium or RPMI 1640 medium supplemented with 10% FBS and 1% penicillin/streptomycin at 37°C in a humidified atmosphere with 5% CO_2_.

### 4.4 Characterization

The morphologies of live Neus and CS-Neus were visualized by SEM (Olympus FV1200). The fluorescent images were captured using a fluorescence microscope (IX73, Olympus) or a confocal laser scanning microscope (A1+, Nikon). Sodium dodecyl sulfate–polyacrylamide gel electrophoresis (SDS-PAGE) and Western blot were run in the Mini-PROTEAN^®^ Tetra Electrophoresis System (Bio-Rad). The Western blotting bands were visualized on a Western blot imaging system (ImageQuant 800, Amersham). The Cell Counting kit 8 (CCK-8) assay and enzyme-linked immunosorbent assay (ELISA) were conducted on a microplate reader (iMark, Bio-Rad). The *in vivo* DiI fluorescence images were captured using an AniView600 multimodal live animal imaging system (Biolight). The average fluorescence intensity of living imaging was calculated using AniView multimodal animal *in vivo* imaging system software and the formula: Average signals = total counts/exposure time (s)/area (pixel).

### 4.5 Neutrophil collection

Fresh human peripheral blood neutrophils were obtained from human donors within 16 h after blood collection. Neutrophils were purified with density gradient centrifugation using the neutrophil isolation solution kit (P9040, Solarbio). In detail, first, 4 mL of reagent A and 2 mL of reagent C were carefully added to the centrifuge tube to form a gradient interface, fresh anticoagulant blood was dispersed over the liquid level of reagent C, and then centrifuged at 750 × g for 25 min. Subsequently, two layers of cells were obtained, with PBMC in the upper layer and neutrophils in the lower layer. Neutrophils were obtained by carefully collecting the cells in the middle of reagent C and reagent A, and in reagent A, the cells were carefully collected into 15-mL clean centrifuge tubes, followed by washing twice with 10 mL of PBS or cell detergent in the kit.

For mouse neutrophils, cells were collected from the whole blood and spleen of ICR mice (eight-week-old, female) using the neutrophil isolation solution kit. Specifically, the blood was collected from the eye frame, and the spleen was collected and ground. Pooled blood and spleen cells were aspirated and placed over a two-layer gradient of reagent A and reagent C. Samples were centrifuged (750 × g, 25 min), and the cell contents from the interface of reagent A and reagent C gradient layers and the lower part of the reagent A layer were collected. RBC lysis buffer was then added to the sample to lyse the erythrocytes, and then, we obtained the neutrophils.

### 4.6 Preparation of CS-Neus

Neutrophils were centrifuged at 300 × g for 5 min and suspended in a non-controlled-rate cell cryopreservation medium at a cell density of 1 × 10^6^ to 1 × 10^7^ mL^−1^. The cell-containing medium was immersed in liquid nitrogen for 12 h to obtain the “cryo-shocked” neutrophils (CS-Neus).

### 4.7 Characterization of CS-Neus

For cell viability analysis, the cells were collected by centrifugation (200 × g, 5 min), washed with PBS, and stained with the Calcein/PI cell viability working fluid for 15–20 min, according to the manufacturer’s protocol. The fluorescent images were captured using a fluorescence microscope (IX73, Olympus). At the same time, the viability of the cells was detected using the Cell Counting Kit-8 (CCK-8, Beyotime). In brief, live Neus and CS-Neus were suspended in the cell culture medium (DMEM, 10% FBS) and added to 96-well plates with a cell density of 8 × 10^3^ per well. After culturing for 0, 4, 8, 12, and 16 h, 10 μL of the Cell Counting kit-8 solution was added to each well. After incubation for 2 h, absorbance was measured at 450 nm using a microplate reader (Bio-Rad).

For cell morphology testing, live Neus and CS-Neus were detected by SEM. To be specific, cells were washed with PBS and fixed with 2.5% glutaraldehyde for at least 12 h. After washing, the cells were dehydrated with ethanol of 20%, 35%, 50%, 70%, 80%, 90%, 95%, and 100% in sequence over 15 min. Finally, the samples were coated by metal spraying and analyzed by SEM (Olympus FV1200).

For the analysis of the cell membrane integrity, the cells were stained using the Cell Plasma Membrane Staining Kit with DiI (Beyotime), according to the manufacturer’s protocol. After staining, the cells were analyzed by confocal microscopy, and the cellular size was measured with Nano Measurer software.

To further analyze the changes after “cryo-shock, ”we examined the cell size and internal structure by flow cytometry. In brief, live Neus and CS-Neus were dispersed at a concentration of 1 × 10^6^ mL^−1^ in PBS and loading at the same voltage.

### 4.8 Secretion of pro-inflammatory cytokines

The cytokine secretion profile was detected through the ELISA assay to examine the pro-inflammatory properties of neutrophils. The live Neus and CS-Neus were incubated with lipopolysaccharides (LPS, 1 μg mL^−1^). The mixtures were reacted for 2 h at 37°C, and the supernatant was collected by centrifugation at 500 × g for 10 min to remove the cells. Cytokines in the supernatant released by neutrophils were quantified by TNF-α and IL-1β enzyme-linked immunosorbent assay (ELISA) kits (Dakewe).

### 4.9 Protein expression of CS-Neus

Whole-cell protein expression was analyzed by SDS-PAGE (sodium dodecyl sulfate–polyacrylamide gel electrophoresis). The proteins were extracted from live Neus and CS-Neus using RIPA lysis (Beyotime) with a protease inhibitor cocktail added (PMSF). Protein concentrations in each group were determined by the BCA assay (Beyotime), according to the manufacturer’s instructions. The samples were denatured with protein loading buffer (Coolaber) for 10 min at 100°C before loading and the quantity of protein in 20 µg per well. The loading samples were electrophoresed in preformed gels (GenScript). After staining with Coomassie brilliant blue, whole-protein imaging was performed.

WB was adopted to detect the expression of pro-inflammatory cytokine receptors (IL-1R, IL-6R, and TNFR), chemokine receptors (CXCR2), and Toll-like receptors (TLR4) in live Neus and CS-Neus. In brief, the proteins of live Neus and CS-Neus were extracted according to the steps of SDS-PAGE gel electrophoresis. The separated protein on the gel was transferred to PVDF membranes and blocked in 5% milk for 2 h. Each band was incubated overnight with primary antibodies (anti-human IL-1R, anti-human IL-6R, anti-human TNFR, anti-human CXCR2, and anti-human TLR4) at 4°C and labeled with the HRP-conjugated secondary antibody the next day. Finally, the image was visualized on a chemiluminescence imager (ImageQuant 800, Amersham).

### 4.10 Transwell migration assay

Cells (live Neus, CS-Neus, PFA-Neus, and Neus without CXCL2, 2 × 10^5^) were added to the top chamber of a transwell and allowed to migrate toward CXCL2 in the bottom chamber. After 6 h at 37°C, cells in the bottom chamber were photographed at different fields of view, and the number of migrated cells in the lower chamber was counted.

### 4.11 Quantification of cytokines and LPS binding

TNF-α (final concentration 600 pg mL^−1^), IL-6 (final concentration 100 pg mL^−1^), or IL-1β (final concentration 400 pg mL^−1^) was mixed with CS-Neus at different final concentrations (10 × 10^4^ mL^−1^, 20 × 10^4^ mL^−1^, 40 × 10^4^ mL^−1^, 80 × 10^4^ mL^−1^, and 160 × 10^4^ mL^−1^) for 6 h at 37°C. The mixtures were centrifuged at 500 × g for 10 min to remove the cells, and the supernatant fluid was collected. The amount of cytokines remaining in the supernatants was then determined by enzyme-linked immunoassay, according to the manufacturer’s protocol. Statistical analysis and non-linear fitting of curves were performed using GraphPad Software version 8. For clinical patients, each patient’s serum was incubated with CS-Neus which were isolated from the same patients for 30 min at 37°C; after centrifugation to remove the cells, the amount of serum remaining cytokines was then measured using the ELISA kit.

To quantify LPS removal with CS-Neus, 10 ng mL^−1^ LPS (Sigma) in 1 × PBS containing 10% FBS was mixed with CS-Neus with varying amounts (10, 20, 40, 80, and 160 × 10^4^ mL^−1^); the free LPS content in the supernatant was determined using the chromogenic endotoxin quantification assay (Thermo Fisher Scientific).

### 4.12 Establishment of LPS-induced subcutaneous local inflammation and the sepsis mouse model

The LPS-induced local inflammation model was established by subcutaneous inoculation of LPS (300 μg mL^−1^, 20 μL) on the right side of the mice back and 20 μL NaCl on the left. The sepsis model was established by an intraperitoneal injection of LPS. Mice were randomly divided into two groups and intraperitoneally injected with NaCl or LPS (30 mg kg^−1^ body weight).

### 4.13 *In vivo* biodistribution of CS-Neus

For the skin local infection model, at 6 h post-infection (h.p.i.), 100 μL of NaCl or DiI-labeled CS-Neus (1.0 × 10^7^ count mL^−1^) were intravenously injected, and fluorescence imaging was performed after 6 h.

For the sepsis model, 200 μL DiI-labeled CS-Neus (2.0 × 10^7^ count mL^−1^) were intravenously injected to healthy mouse and sepsis mouse at 12 h post-LPS injection. At 1, 6, and 12 h after injection, fluorescence images of their vital organs (the heart, liver, spleen, lung, and kidney) were recorded on the AniView100 multimodal live animal imaging system (BLT) with an excitation wavelength of 549 nm. To observe the fluorescence intensity of lung tissue, their frozen lung slices were prepared and stained with DAPI, and then, the lung slides were observed with fluorescence microscopy. The average fluorescent intensity of lung tissues was calculated using AniView multimodal animal *in vivo* imaging system software.

### 4.14 *In vivo* anti-inflammatory experiments

Female ICR mice (8–12 weeks old) were randomly divided into four groups (sham, untreated, live Neu, and CS-Neu, six mice per group). At 2, 4, and 6 h, 9.0 × 10^6^ count of live Neus or CS-Neus were injected intravenously. The survival rate and their body temperature were recorded at 0, 6, 12, and 24 h. At 48 h after model construction, mice were sacrificed, and the blood serum and vital organs from mice were harvested for cytokine mRNA expression level analysis, prepared as paraffin sections, and stained with H&E using standard procedures.

### 4.15 Histological study

Mice were sacrificed after different treatments, and the collected organs were perfused with 4% paraformaldehyde (PFA), and gradient dehydration was performed in alcohol, followed by paraffin embedding. The fixed organs were cut into 5 µm sections and then stained with H&E and Masson for pathology analysis (Wang et al., 2013).

### 4.16 Biosafety assessment

For *in vitro* toxicity evaluation, different concentrations of CS-Neus (10 × 10^5^, 20 × 10^5^, 50 × 10^5^, 100 × 10^5^, and 200 × 10^5^ mL^−1^) were incubated with two types of normal cells (L929 and HUVEC). After 24 h of co-incubation, the proliferation and viability of these treated cells were assayed by the CCK-8 kit (Beyotime Biotechnology). For *in vivo* toxicity analysis, healthy female ICR mice (8–12 weeks old, three for each group) were intravenously injected with 250 μL of NaCl or CS-Neus (4 × 10^7^ count mL^−1^, twice that of *in vivo*). The blood was collected through the eye, and routine blood test indices of mice including red blood cells (RBCs), hemoglobin (HGB), white blood cells (WBCs), and platelets (PLTs) were carried out. To estimate biomarker levels associated with the kidney and liver, liver function indexes including alanine transaminase (ALT), aspartate aminotransferase (AST), and albumin (ALB) and renal function indexes including creatinine (CRE) and blood urea nitrogen (BUN) were analyzed. In addition, vital organs (the heart, liver, spleen, lung, and kidney) of mice were collected for histological analysis.

### 4.17 Statistical analysis

Quantitative data in this study were expressed as the mean ± standard deviation (SD) in three or more independent experiments. Statistical significance was calculated through Student’s t-test, ordinary one-way ANOVA, or a log-rank (Mantel–Cox) test and performed using GraphPad Software version 8. *p < 0.05 was regarded as statistically significant. *p < 0.05; **p < 0.01; and ***p < 0.001.

## Data Availability

The original contributions presented in the study are included in the article/Supplementary Material; further inquiries can be directed to the corresponding authors.
